# Supplementary material for: King’s Sarcoidosis Questionnaire (KSQ) – Validation study in Serbian speaking population of sarcoidosis patients
Source: PLoS One. 2023 Sep 5;18(9):e0273126. doi: 10.1371/journal.pone.0273126 (PMC10479938; doi:10.1371/journal.pone.0273126)
Supplement: S2 File — (DOCX) [file pone.0273126.s002.docx]

| In the last 2 weeks… | **All of the time** | **Most of the time** | **A good bit of the time** | **Some of the time** | **A little bit of the time** | **Hardly any of the time** | **None of the time** |
| --- | --- | --- | --- | --- | --- | --- | --- |
| **Section 1:**  **General Health** |  | | | | | | |
| I have felt frustrated |  |  |  |  |  |  |  |
| I have had trouble concentrating |  |  |  |  |  |  |  |
| I have lacked motivation |  |  |  |  |  |  |  |
| I have felt tired |  |  |  |  |  |  |  |
| I have felt anxious |  |  |  |  |  |  |  |
| I have felt aches and pains in my muscles/joints |  |  |  |  |  |  |  |
| I have felt embarrassed |  |  |  |  |  |  |  |
| I have worried about my weight |  |  |  |  |  |  |  |
| I have worried about my sarcoidosis |  |  |  |  |  |  |  |
| Tiredness has interfered with my normal social activities such as going out with friends/family |  |  |  |  |  |  |  |
| **Section 2: Lung** |  | | | | | | |
| My cough has caused pain/discomfort |  |  |  |  |  |  |  |
| I have been breathless climbing stairs or walking up slight inclines |  |  |  |  |  |  |  |
| I have had to take deep breaths, also known as ‘air hunger’ |  |  |  |  |  |  |  |
| My chest has felt tight |  |  |  |  |  |  |  |
| I have had episodes of breathlessness |  |  |  |  |  |  |  |
| I have experienced chest pains |  |  |  |  |  |  |  |
| **Section 3: Medication** |  | | | | | | |
| I have worried about side effects of my medication for sarcoidosis |  |  |  |  |  |  |  |
| I have felt worse because of my medication |  |  |  |  |  |  |  |
| I have gained weight because of my medication |  |  |  |  |  |  |  |
| **Section 4: Skin** |  | | | | | | |
| I have been bothered by my skin problems |  |  |  |  |  |  |  |
| I have been concerned about changes in colour of my skin lesions |  |  |  |  |  |  |  |
| I have been embarrassed about my skin |  |  |  |  |  |  |  |
| **Section 5: Eyes** |  | | | | | | |
| I have had dry eyes |  |  |  |  |  |  |  |
| I have had difficulty with bright lights |  |  |  |  |  |  |  |
| My eyes have been red |  |  |  |  |  |  |  |
| I have had pain in/or around the eyes |  |  |  |  |  |  |  |
| I have had difficulty reading |  |  |  |  |  |  |  |
| I have had blurred vision |  |  |  |  |  |  |  |
| I have been worried about my eyesight |  |  |  |  |  |  |  |
