# Supplementary material for: King’s Sarcoidosis Questionnaire (KSQ) – Validation study in Serbian speaking population of sarcoidosis patients
Source: PLoS One. 2023 Sep 5;18(9):e0273126. doi: 10.1371/journal.pone.0273126 (PMC10479938; doi:10.1371/journal.pone.0273126)
Supplement: S3 File — (DOCX) [file pone.0273126.s003.docx]

Kingov test za sarkoidoze- Srpska verzija

U poslednje 2 nedelje, ostajao/la sam bez daha dok sam se peo/la stepenicama ili na uzbrdici.

1.Uvek 2.Većinu vremena 3.Nekoliko puta 4.Ponekad 5.S vremena na vreme 6.Retko 7.Nikad

U poslednje 2 nedelje, zbog mog stanja sa plućima, osetio/la sam pritisak u grudima.

1.Uvek 2.Većinu vremena 3.Nekoliko puta 4.Ponekad 5.S vremena na vreme 6.Retko 7.Nikad

U poslednje 2 nedelje, da li ste brinuli o ozbiljnosti Vašeg stanja?

1.Uvek 2.Većinu vremena 3.Nekoliko puta 4.Ponekad 5.S vremena na vreme 6.Retko 7.Nikad

U poslednje 2 nedelje, da li ste izbegavali stvari zbog kojih ostajete bez daha?

1.Uvek 2.Većinu vremena 3.Nekoliko puta 4.Ponekad 5.S vremena na vreme 6.Retko 7.Nikad

U poslednje 2 nedelje, da li ste osećali da imate kontrolu nad Vašim plućnim oboljenjem?

1.Uvek 2.Većinu vremena 3.Nekoliko puta 4.Ponekad 5.S vremena na vreme 6.Retko 7.Nikad

U poslednje 2 nedelje, da li je Vaša plućna bolest izazvala u Vama osećaj da Vam je dosta svega?

1.Uvek 2.Većinu vremena 3.Nekoliko puta 4.Ponekad 5.S vremena na vreme 6.Retko 7.Nikad

U poslednje 2 nedelje, imao/la osećaj gladi za vazduhom.

1.Uvek 2.Većinu vremena 3.Nekoliko puta 4.Ponekad 5.S vremena na vreme 6.Retko 7.Nikad

U poslednje 2 nedelje, moja plućna bolest mi je izazvala napad panike.

1.Uvek 2.Većinu vremena 3.Nekoliko puta 4.Ponekad 5.S vremena na vreme 6.Retko 7.Nikad

U poslednje 2 nedelje, koliko često ste osetili „zviždanje“ iz grudi?

1.Uvek 2.Većinu vremena 3.Nekoliko puta 4.Ponekad 5.S vremena na vreme 6.Retko 7.Nikad

U poslednje 2 nedelje, koliko često ste smatrali da se Vaša plućna bolest pogoršava?

1.Uvek 2.Većinu vremena 3.Nekoliko puta 4.Ponekad 5.S vremena na vreme 6.Retko 7.Nikad

U poslednje 2 nedelje, koliko često je Vaša plućna bolest izazvala smanjeno svakodnevno funkcionisanje ili otežala Vaš posao?

1.Uvek 2.Većinu vremena 3.Nekoliko puta 4.Ponekad 5.S vremena na vreme 6.Retko 7.Nikad

U poslednje 2 nedelje, koliko često se Vaša plućna bolest pogoršala?

1.Uvek 2.Većinu vremena 3.Nekoliko puta 4.Ponekad 5.S vremena na vreme 6.Retko 7.Nikad

U poslednje 2 nedelje, koliko često je Vaša bolest ograničila Vašu sposobnost svakodnevnog nošenja stvari, npr. namernica?

1.Uvek 2.Većinu vremena 3.Nekoliko puta 4.Ponekad 5.S vremena na vreme 6.Retko 7.Nikad

U poslednje 2 nedelje, koliko puta je Vaša plućna bolest dovela do toga da razmišljate o smrti?

1.Uvek 2.Većinu vremena 3.Nekoliko puta 4.Ponekad 5.S vremena na vreme 6.Retko 7.Nikad

Da li ste u finansijski goroj situaciji kao posledica Vaše bolesti?

1.Izuzetno značajno 2.Značajno 3.Umereno značajno 4.Ne toliko značajno 5.Malo 6.Gotovo ništa 7.Ništa
