# Supplementary material for: King’s Sarcoidosis Questionnaire (KSQ) – Validation study in Serbian speaking population of sarcoidosis patients
Source: PLoS One. 2023 Sep 5;18(9):e0273126. doi: 10.1371/journal.pone.0273126 (PMC10479938; doi:10.1371/journal.pone.0273126)
Supplement: Attachment — Submitted filename: (1) PONE-D-22-20583.pdf [file pone.0273126.s004.pdf]

PLOS ONE

King’s Sarcoidosis Questionnaire (KSQ) – Validation study in non-English speaking population of sarcoidosis patients

--Manuscript Draft--

Manuscript Number:	PONE-D-22-20583
Article Type:	Research Article
Full Title:	King’s Sarcoidosis Questionnaire (KSQ) – Validation study in non-English speaking population of sarcoidosis patients
Short Title:	Serbian version of KSQ
Corresponding Author:	Mihailo Stjepanovic<br>University of Belgrade, Medical Faculty<br>Belgrade, SERBIA
Keywords:	sarcoidosis; Quality of life; KSQ; validation
Abstract:	Sarcoidosis is a multiorgan, multisystem chronic disease of unknown etiology and unpredictable course. Health status is reduced in sarcoidosis and assessing it is a difficult multitask effort due to many faces this disease might have. Despite the studies performed with the aim to find out the reliable tools for assessment the disease severity or its progression, up to date there is no reliable tool for assessing the impact of sarcoidosis on patients’ health status and the impact of the therapy. At the time of writing, the most used disease specific questionnaire for assessing quality of life in sarcoidosis was Sarcoidosis health Questionnaire, created by Christopher Cox and coworkers. Recently, a new questionnaire for assessing health status in sarcoidosis was developed by a group of authors from England – King’s Sarcoidosis Questionnaire (KSQ). The benefit of KSQ is the ability to develop the best care plan for the patient, as well as to differentiate the efficacy of the administered treatment.<br>Objective<br>The aim of this study was to validate the KSQ in non–English speaking European population of sarcoidosis patients. The test itself is a modular, multi-organ health status measure for patients with sarcoidosis for use in clinic and the evaluation of therapies. The correlation of KSQ with different clinical course of sarcoidosis (acute vs chronic disease) and with the clinical outcome status (COS) in sarcoidosis was also investigated.<br>Methods<br>A total of 159 biopsy positive sarcoidosis patients participated in this study. The average age of the participants was 49.67, majority was female (67.3%) and majority had only pulmonary form of sarcoidosis (71.7%). KSQ - new disease-specific health status instrument, was compared with 5 other already existing instruments already used and validated in sarcoidosis (Saint George Respiratory Questionnaire- SGRQ, Daily Activity List -DAL, Fatigue Assessment Scale- FAS, Medical Research Council dyspnea scale– MRC, Borg Dyspnea Scale and 15D as general questionnaire.<br>Results<br>KSQ has significant correlation with other quality of life questionnaires already used in sarcoidosis. Translated, validated and culturally adapted version of KSQ shows significant internal reliability, similar to the original KSQ. Serbian version of KSQ has significant correlation with different clinical course of sarcoidosis and with COS as well. The translated version of KSQ is reliable sarcoidosis specific instrument for assessing health status in these patients.
Order of Authors:	Violeta Mihailovic-Vucinic
	Mihailo Stjepanovic
	Branislav S. Gvozdenovic
	Jelena Milin-Lazovic
	Slobodan Belic
	Natasa Djurdjevic

Powered by Editorial Manager® and ProduXion Manager® from Aries Systems Corporation

	Nikola Maric
	Aleksa Golubovic
**Additional Information:**	
**Question**	**Response**
**Financial Disclosure**<br><br>Enter a financial disclosure statement that describes the sources of funding for the work included in this submission. Review the submission guidelines for detailed requirements. View published research articles from *PLOS ONE* for specific examples.<br><br>This statement is required for submission and **will appear in the published article** if the submission is accepted. Please make sure it is accurate.<br><br>**Unfunded studies**<br>Enter: *The author(s) received no specific funding for this work.*<br><br>**Funded studies**<br>Enter a statement with the following details:<br>• Initials of the authors who received each award<br>• Grant numbers awarded to each author<br>• The full name of each funder<br>• URL of each funder website<br>• Did the sponsors or funders play any role in the study design, data collection and analysis, decision to publish, or preparation of the manuscript?<br>• **NO** - Include this sentence at the end of your statement: *The funders had no role in study design, data collection and analysis, decision to publish, or preparation of the manuscript.*<br>• **YES** - Specify the role(s) played.<br><br>* typeset	The author(s) received no specific funding for this work.
**Competing Interests**<br><br>Use the instructions below to enter a competing interest statement for this submission. On behalf of all authors, disclose any competing interests that could be perceived to bias this work—acknowledging all financial support and any other relevant financial or non-	The authors have declared that no competing interests exist.

financial competing interests.<br><br>This statement is required for submission and **will appear in the published article** if the submission is accepted. Please make sure it is accurate and that any funding sources listed in your Funding Information later in the submission form are also declared in your Financial Disclosure statement.<br><br>View published research articles from *PLOS ONE* for specific examples.<br><br>**NO authors have competing interests**<br><br>Enter: *The authors have declared that no competing interests exist.*<br><br>**Authors with competing interests**<br><br>Enter competing interest details beginning with this statement:<br><br>*I have read the journal's policy and the authors of this manuscript have the following competing interests: [insert competing interests here]*<br><br>* typeset	
**Ethics Statement**<br><br>Enter an ethics statement for this submission. This statement is required if the study involved:<br><br>• Human participants<br>• Human specimens or tissue<br>• Vertebrate animals or cephalopods<br>• Vertebrate embryos or tissues<br>• Field research<br><br>Write "N/A" if the submission does not require an ethics statement.<br><br>General guidance is provided below. Consult the submission guidelines for	Ethic board University clinical center of Serbia; no. 527/22<br>Written consent was obtained form the participants.

detailed instructions. **Make sure that all information entered here is included in the Methods section of the manuscript.**<br><br>**Format for specific study types**<br><br>**Human Subject Research (involving human participants and/or tissue)**<br>• Give the name of the institutional review board or ethics committee that approved the study<br>• Include the approval number and/or a statement indicating approval of this research<br>• Indicate the form of consent obtained (written/oral) or the reason that consent was not obtained (e.g. the data were analyzed anonymously)<br><br>**Animal Research (involving vertebrate animals, embryos or tissues)**<br>• Provide the name of the Institutional Animal Care and Use Committee (IACUC) or other relevant ethics board that reviewed the study protocol, and indicate whether they approved this research or granted a formal waiver of ethical approval<br>• Include an approval number if one was obtained<br>• If the study involved *non-human primates*, add *additional details* about animal welfare and steps taken to ameliorate suffering<br>• If anesthesia, euthanasia, or any kind of animal sacrifice is part of the study, include briefly which substances and/or methods were applied<br><br>**Field Research**<br><br>Include the following details if this study involves the collection of plant, animal, or other materials from a natural setting:<br>• Field permit number<br>• Name of the institution or relevant body that granted permission	
**Data Availability**<br><br>Authors are required to make all data underlying the findings described fully available, without restriction, and from the time of publication. PLOS allows rare	Yes - all data are fully available without restriction

exceptions to address legal and ethical concerns. See the PLOS Data Policy and FAQ for detailed information.<br><br>A Data Availability Statement describing where the data can be found is required at submission. Your answers to this question constitute the Data Availability Statement and **will be published in the article**, if accepted.<br><br>**Important:** Stating 'data available on request from the author' is not sufficient. If your data are only available upon request, select 'No' for the first question and explain your exceptional situation in the text box.<br><br>Do the authors confirm that all data underlying the findings described in their manuscript are fully available without restriction?	
**Describe where the data may be found in full sentences. If you are copying our sample text, replace any instances of XXX with the appropriate details.**<br><br>• If the data are **held or will be held in a public repository**, include URLs, accession numbers or DOIs. If this information will only be available after acceptance, indicate this by ticking the box below. For example: *All XXX files are available from the XXX database (accession number(s) XXX, XXX.*).<br>• If the data are all contained **within the manuscript and/or Supporting Information files**, enter the following: *All relevant data are within the manuscript and its Supporting Information files.*<br>• If neither of these applies but you are able to provide **details of access elsewhere**, with or without limitations, please do so. For example:<br><br>*Data cannot be shared publicly because of [XXX]. Data are available from the XXX Institutional Data Access / Ethics Committee (contact via XXX) for researchers who meet the criteria for*	All relevant data are within the manuscript and its Supporting Information files.

access to confidential data.<br><br>*The data underlying the results presented in the study are available from (include the name of the third party and contact information or URL).*<br>• This text is appropriate if the data are owned by a third party and authors do not have permission to share the data.<br><br>* typeset	
Additional data availability information:	Tick here if the URLs/accession numbers/DOIs will be available only after acceptance of the manuscript for publication so that we can ensure their inclusion before publication.

Powered by Editorial Manager® and ProduXion Manager® from Aries Systems Corporation

Manuscript Click here to access/download;Manuscript;KING 20072022.docx

King's Sarcoidosis Questionnaire (KSQ) – Validation study in non-English speaking population of sarcoidosis patients

Violeta Mihailovic-Vucinic[1¶], Mihailo Stjepanovic[1,2¶*], Branislav S. Gvozdenovic[3¶], Jelena Milin-Lazovic[4¶], Slobodan Belic[1¶], Natasa Djurdjevic[1¶], Nikola Maric[1¶]

[1] Clinic of pulmonology, University clinical center of Serbia, Belgrade, Serbia

[2] Faculty of Medicine, University of Belgrade, Belgrade, Serbia

[3] PPD Serbia, Pharmacovigilance Department, Belgrade, Serbia

[4] Institute for Medical Statistics and Informatics, Faculty of Medicine, University of Belgrade, Belgrade, Serbia.

* Corresponding author

e-mail: mihailostjepanovic@gmail.com (MS)

[¶]These authors contributed equally to this work.

ABSTRACT

Introduction

Sarcoidosis is a multiorgan, multisystem chronic disease of unknown etiology and unpredictable course. Health status is reduced in sarcoidosis and assessing it is a difficult multitask effort due to many faces this disease might have. Despite the studies performed with the aim to find out the reliable tools for assessment the disease severity or its progression, up to date there is no reliable tool for assessing the impact of sarcoidosis on patients' health status and the impact of the therapy. At the time of writing, the most used disease specific questionnaire for assessing quality of life in sarcoidosis was Sarcoidosis health Questionnaire, created by Christopher Cox and coworkers. Recently, a new questionnaire for assessing health status in sarcoidosis was developed by a group of authors from England – King's Sarcoidosis Questionnaire (KSQ). The benefit of KSQ is the ability to develop the best care plan for the patient, as well as to differentiate the efficacy of the administered treatment.

Objective

The aim of this study was to validate the KSQ in non–English speaking European population of sarcoidosis patients. The test itself is a modular, multi-organ health status measure for patients with sarcoidosis for use in clinic and the evaluation of therapies. The correlation of KSQ with different clinical course of sarcoidosis (acute vs chronic disease) and with the clinical outcome status (COS) in sarcoidosis was also investigated.

Methods

A total of 159 biopsy positive sarcoidosis patients participated in this study. The average age of the participants was 49.67, majority was female (67.3%) and majority had only pulmonary form of sarcoidosis (71.7%). KSQ - new disease-specific health status instrument, was compared with 5 other already existing instruments already used and validated in sarcoidosis (Saint George Respiratory Questionnaire- SGRQ, Daily Activity List -DAL, Fatigue Assessment Scale- FAS, Medical Research Council dyspnea scale– MRC, Borg Dyspnea Scale and 15D as general questionnaire.

Results

KSQ has significant correlation with other quality of life questionnaires already used in sarcoidosis. Translated, validated and culturally adapted version of KSQ shows significant internal reliability, similar to the original KSQ. Serbian version of KSQ has significant correlation with different clinical course of sarcoidosis and with COS as well. The translated

version of KSQ is reliable sarcoidosis specific instrument for assessing health status in these patients.

Keywords: sarcoidosis, quality of life, KSQ, validation

Introduction

Sarcoidosis is a multisystem granulomatous disease of unknown etiology and unpredictable course. Granulomatous inflammation can affect any organ in the body and the clinical manifestations of sarcoidosis vary in relation to the affected organs [1].

Pulmonary involvement is present in a vast majority of patients, so the evaluation of pulmonary function tests (PFTs) and chest X ray findings are frequently used in daily practice to assess the disease severity [2]. However, these tests often poorly correlate with patients` symptoms. Studies evaluating these problems showed poor agreement between physicians and patients regarding the perceived symptoms of sarcoidosis [3]. The gap is even bigger regarding the failure of physicians to evaluate the impact of non-specific features of sarcoidosis related to sensation of fatigue and cognitive function impairments which evidently do occur in sarcoidosis [4,5].

Sarcoidosis usually affects population of working age, who may find very difficult to cope with this disease and its consequent and certainly negative impact on their health status and quality of life. Despite the studies performed with the aim to find out the reliable tools for assessment the disease severity or its progression, up to date there is no reliable tool for assessing the impact of sarcoidosis on patients' health status, and also the impact of therapy with all possible side effects.

Until now the only developed disease-specific questionnaire for measuring the health status in sarcoidosis was the Sarcoidosis Health Questionnaire, created by Christopher Cox and coworkers; it exanimated three domains: Daily Functioning, Physical Functioning, and Emotional Functioning [6]. This questionnaire was validated in patients attending a university medical center in the US where 80% of them were African-Americans [6].

Recently new questionnaire for assessing the health status in sarcoidosis was developed by a group of authors from England: The King's Sarcoidosis Questionnaire (KSQ) [7]. The KSQ has 5 sections: general health status, lungs, medication, skin and eyes.

The aim of this study was to validate the KSQ in non-English speaking population of sarcoidosis patients. The objective was also to compare the KSQ as disease-specific health status questionnaire with other already used questionnaires in sarcoidosis.

Methods and patients

The cross-sectional study was conducted in the Clinic for Pulmonology of the Clinical Center of Serbia in Belgrade over the period of 6 months.

Patients

We enrolled 159 biopsy positive sarcoidosis patients diagnosed in the Clinic. They all had noncaseating epithelioid cell granulomas on their lung biopsy specimens, without a known cause being identified. All subjects were 18 years old and older, and did not have any associated illnesses that could influence their health status (those with significant comorbidity, like cardiac disorders, were excluded from analysis). Subjects who did not understand the questions from any of the symptoms or health status administered questionnaires were also excluded. All patients were examined during regularly scheduled clinical visits.

Demographic data was collected, with classification of current organ involvement based on the ACCESS study [8].

This study was approved by the institution's ethics committee and all patients consented to participation.

Besides the demographic data, we also collected pulmonary function tests, other, previously validated tests, and the outcome of sarcoidosis.

Pulmonary function tests

On the same day patients completed the KSQ, performed spirometry tests on a pneumotachograph (Jaeger, Germany) with measures expressed as % of the reference values according to the ATS/ERS criteria [9]. Pulmonary function measurements included forced expiratory vital capacity (FVC), forced expiratory volume in 1 second (FEV_1), peak expiratory flow (PEF). The transfer factor of the lung for carbon monoxide (DLCO) was measured using the single-breath method (Masterlab, Jaeger, Wurzburg, Germany).

Questionnaires used in this study

During the regularly scheduled outpatient clinic visit patients completed King`s Sarcoidosis Questionnaire (KSQ) and also two standardized questionnaires for the measuring of health status: a generic measure – the fifteen-dimensional measure of health-related quality of life (15D) [10] and the Saint George Respiratory Questionnaire (SGRQ) [11].

15D is an instrument for measurement of health-related quality of life [10]. It was developed and validated in a large Finnish population. It consists of 15 different and mutually exclusive health dimensions, each represented by one item [12,13]. The total questionnaire score

ranges between 0 and 1, where 1 signifies the highest level of health status. 15D was used in different diseases in many different countries. The Serbian version of 15D was previously used in patients with asthma where it demonstrated good psychometric measurement properties [15].

SGRQ is an instrument that was originally designed to measure the health status of COPD patients [11]. Its validity, reliability, and responsiveness were also shown in other pulmonary diseases and sarcoidosis too [15]. The questionnaire consists of 50 items with 76 responses, and encompasses three domains of health status: 1) symptoms, focusing on distress because of respiratory symptoms; 2) activities, measuring decreased mobility or physical activity and 3) impacts, measuring the psychosocial influence of disease on the everyday life and patients' well-being. Scores of these domains, as well as the total score, are scaled from 0 to 100, where higher scores represent poorer health status.

Dyspnea questionnaire

Dyspnea was measured by the Modified Medical Research Council (MRC) Dyspnea Scale [16]. The MRC scale classifies subjects into one of five categories according to their degree of dyspnea when performing certain activities. Scores range from 0 to 4, with the higher scores indicating more severe dyspnea.

Activities of daily living

The degree of limitation in activities of daily living was evaluated with the List of Daily Activities (DAL), a scale that was originally designed by Stewart and coworkers [17]. DAL has 11 items that are related to the usual activities that persons with good health can perform without particular effort. The number of positive responses comprises the DAL score and indicates the degree of impairment. The scale has been used in several studies in patients with chronic pulmonary diseases. DAL has been validated in serbian sarcoidosis patients as well [18,19].

Fatigue measurement

Fatigue Assessment Scale (FAS) was used to assess sensation of fatigue and correlate it with KSQ as a potential measure for fatigue in sarcoidosis. FAS consists of 10 items: five questions reflecting physical fatigue and five questions for mental fatigue. The response

scale is a 5-point scale (1 - never to 5 - always). Scores on FAS can range from 10-50. FAS scores < 22 indicate no fatigue.

All questionnaires except FAS, were already used in assessing symptom severity in our population of patients with sarcoidosis [19]. However, FAS has been validated in a broad spectrum of fatigue studies in sarcoidosis [4,5]. FAS shows good validity and reliability in sarcoidosis patients and measures fatigue as distinct from depression [4,15,20,21].

Clinical outcome of sarcoidosis

The clinical outcome of sarcoidosis is quite variable. Several scoring systems have been used to assess the level of disease and clinical outcome. WASOG (World Association of Sarcoidosis and other Granulomatous diseases) task force was to define clinical phenotypes of disease based on clinical outcome in sarcoidosis patients (COS). To determine COS category, it is mandatory that the patients are followed up for five years after the diagnosis was established [22].

The components of sarcoidosis were incorporated into the final nine COS categories, including the following: the current or past need for systemic therapy, the resolution of the disease, and current status of the condition. The estimated COS categories are:

Resolved: COS 1- Never treated, COS 2- No therapy > One year;

Minimal Disease: COS 3 - Never treated, COS 4 - No therapy > One year;

Persistent sarcoidosis with no current therapy: COS 5- Never treated, COS 6 - No therapy > One year;

Persistent sarcoidosis with current therapy: No worsening prior year: COS 7- Asymptomatic, COS 8- Symptomatic,

Persistent sarcoidosis with current therapy: COS 9 - Worsening in prior year

KSQ Questionnaire

King’s Sarcoidosis Questionnaire (KSQ) is a modular multi-organ health status measure for patients with sarcoidosis for use in clinic and the evaluation of therapies. It consists of five modules: General health status-GHS (10 items), Lung (6 items), Medication (3 items), Skin (3 items) and Eye (7 items). The General health status module is intended to be administered to all patients with sarcoidosis. In addition to this, patients also complete organ specific modules if relevant to their condition.

The individual module scores are intended to identify the health domains affected.

The medication module can be used in isolation or combined with overall lung and skin health status questionnaires but not eye health status.

Patients complete the original seven-point Likert scale and scoring is calculated using a re-ordered scale for appropriate items. The KSQ module and overall (total) scores were transformed to a range of 0–100 [(actual score − lowest possible score/range) ×100], where 100 represents the best health status [7].

Questionnaire translation process

The original English version of the KSQ was officially translated and adopted into the Serbian language with the assistance of two mother tongue experts who worked independently in the translation from English to Serbian and back translation from Serbian to English. All items from the questionnaire were standardized for validity once again in collaboration with the sarcoidosis experts from the Serbian Association of Sarcoidosis (SAS).

The backward version of the KSQ translation was emailed to the authors of the original KSQ (S Birring and A Patel), so they could give the permission for its use.

The KSQ scores for this study were calculated by the coauthor of the KSQ study, Dr Amit Patel.

After obtaining the final Serbian version, cognitive debriefing was performed; five patients and five physicians were asked if they completely understand the questions from the KSQ. In addition, we asked both doctors and patients to write down their impressions about the new questionnaire. Furthermore, the doctors were asked to give a mark for the questionnaire at the scale from 0-5.

Here are doctors` comments about the KSQ, together with the marks.

Doctors' comments about KSQ:

Milica Kontic, MD, PhD

I think that KSQ is clear, specific and very well formulated and that will be understandable to sarcoidosis patients and easy to fill out.

(5)

Ana Blanka, MD

I think that questionnaire is very good, because covers all aspects of sarcoidosis. Questions are clear, short and precise which makes this questionnaire understandable and easy to answer. Overview (appearance) is also very clear.

(5)

Jasmina Maric Zivkovic, MD, PhD

Symptoms from all sections are comprehensively (overall) presented. The questionnaire its self is very clear. The only remark: I would change is the order of questions in lung, medication and skin section. In medication and lung section the last question should be in the first place. I think that this sarcoidosis questionnaire is very good and useful. It’s not very long and our patients shouldn’t have any difficulties answering proposed questions.

(5)

Snezana Raljevic, MD

Questionnaire consists of 29 questions, divided into 5 subgroups: General health status, lungs, medications, skin, eyes. Questionnaire is short and clear. It wouldn’t’t take too long for a patient to answer. Questions are understandable to patients and almost all conditions were included that could affect quality of life of sarcoidosis patient. Questions and answers are very well designed so the patients themselves could fill out this questionnaire without any help from investigators.

My opinion is positive for KSQ and I would recommend it for use.

(5)

Aleksandra Dudvarski-Ilic, MD, PhD

Questions absolutely reflect the patients’ opinion about their disease. Almost all aspects of sarcoidosis are represented in this questionnaire that are important for patients. KSQ ic clear and overview is very good. Some terms in Serbian language could be more adjusted.

(4)

Patients about KSQ

Here we also considered patients` medical history , sarcoidosis duration and organ involvement. Additionally, patients were asked to give a mark for the questionnaire at the scale from 0-5.

O.M/ Age 31, sarcoidosis duration: 1-year, Current therapy: Prednisone

I think that this questionnaire includes most of the problems that sarcoidosis patients encounter. It’s very clear and easy to answer.

(5)

I.Dj/ Age 50, sarcoidosis duration: 1-year, Current therapy: No

This questionnaire covers all symptoms of our disease, and I didn't have any difficulties answering these questions. I am also pleased that there are people who are trying to make our disease easier in this way.

(5)

S.D/Age74, sarcoidosis duration: 8 years, Current therapy: Prednisone+ Methotrexate

I have read this questionnaire very carefully and I think that includes a lot of symptoms that are related to sarcoidosis.

(5)

S. P/Age 56, sarcoidosis duration: 4 years, Current therapy: No

I think that based on my course of the disease this questionnaire covered the essence of sarcoidosis and all the symptoms and conditions sarcoidosis patient can experience.

(5)

D.J/Age 42, sarcoidosis duration: 5 years, Current therapy: Prednisone+ Methotrexate

Questions are precise, but I had hard time answering them. I believe the main lack of this questionnaire is that questions only consider 2-week period. I believe so, because while I am in a hospital, I rest all day and my symptoms are less there comparing when I am at home and having all my daily routine activities. I think real answers could be given when I am in my everyday environment.

(3)

Statistical analysis

Descriptive statistics were calculated for the baseline demographic and clinical features. Continuous variables were presented as means with standard deviations and 95% confidence intervals (CI), while categorical variables are presented with numbers and percentages. Normality of distribution for continuous variables was tested with mathematical and graphical methods.

Construct validity between the general and organ specific domains of KSQ and the corresponding questionnaires were determined using Spearman's correlation coefficients. The internal consistency of the Serbian version of KSQ was assessed for multiple item scales by using Cronbach's alpha coefficient (ranges from 0–1, the latter meaning perfect reliability).

Differences between KSQ scores in groups with acute and chronic sarcoidosis were analyzed using Students t-test (or Mann Whitney test) as appropriate. Correlation between KSQ scores

with COS score, other questionnaires scores and lung functions were calculated using Pearson (or Spearman) correlation coefficient as appropriate.
The level of significance was set at 0.05. Statistical analysis was performed using the IBM SPSS 21 (Chicago, IL, 2012) package.

Results

Construct validity between the general and organ specific domains of KSQ and the corresponding questionnaires are presented in **Table 1.** Correlation between KSQ modules and SGRQ domains, DAL MRC, FAS and 15D scores were mostly moderate to strong, negative and statistically significant, except for medication and skin score. The correlations between the KSQ GHS and lung domain and all questionnaires (SGRQ domains, DAL MRC, FAS and 15D) were negative, moderate to strong (r= -0.523 to -0.751). KSQ medication and skin score showed a negative, weak to moderate correlation with SGRQ domains, DAL MRC, FAS and 15D (r= -0.175 to - 0.350). The Eyes module showed a negative, moderate to strong correlation with the other questionnaires (r=-0.382 to -0.574). KSQ organ modules combined with the GHS module all showed a moderate to strong correlation with the SGRQ domains, DAL MRC, FAS and 15D.
Internal reliability for KSQ translated version and original version are showed in **Table 2.** Cronbach's α coefficients for lungs, skin and eye were higher in translated version of KSQ. Cronbach's α for general health status and medication were lower in Serbian version of KSQ, when compare to original version.
Average age for patients with sarcoidosis was 49.67±11.12 and 2/3 were female. The baseline characteristics of the 159 patients who participated in the study are shown in **Table 3.** Patients with chronic sarcoidosis showed lower KSQ scores in general (**Table 4**).
Patients with chronic sarcoidosis showed statistically significant lower scores for all KSQ modules except for GHS and medication score. Also, GHS lung score was lower in chronic patients, difference was close to conventional level of significance.
For patients with follow up period more than 5 years, clinical outcome status (COS) was analyzed as they were classified into 9 categories (**Table 5**).
Correlation between lung function parameters, clinical outcome of sarcoidosis and KSQ scores correlation are presented in **Table 6.** There was no statistically significant correlation between FVC and KSQ modules. FEV 1 correlate positive statistically significant only with

lung score. Positive, statistically significant correlation was observed between PEF and DLco with GHS, Lung, Eye, GHS Lung, GHS Skin score, GHS Eye, GHS Lung Skin, GHS Lung Medication, GHS Skin Medication and GHS Lung Skin Medication score. Statistically significant negative correlation between COS score and KSQ lung, GSH Lung, GSH S, GSH LM, GSH SM, GSH LMS were observed. Negative, moderate statistically significant correlation was observed between COS score and medication score. Correlation between COS and GSH score was negative and close to conventional level of significance. There was no statistically significant correlation between skin, eyes, GHS Eye and COS score.

Discussion

Sarcoidosis is multiorgan, multisystem disease affecting patients` health in many different ways. The relationship between HS and sarcoidosis was first examined in 1997 [23]. Since that time until now authors have been trying to find out the best measure considering patients quality of life and /or health status [24].

Health status is reduced in sarcoidosis and assessing it is a difficult multitask effort due to many faces the disease might have. The clinical course of sarcoidosis is unpredictable. More than 90% of sarcoidosis patients have lung disease. However, other organs like the lymph nodes, skin, and eyes are frequently involved. Patients with pulmonary sarcoidosis are often presented with respiratory symptoms while other general symptoms such as fever, anorexia, weight loss, pain and fatigue are present as well. All of the above is disabling for the patient and cause an impaired quality of life.

Numerous studies showed that fatigue is an important feature of sarcoidosis [25-28].

Considering the health status evaluation, several generic and disease-specific measures have been used in sarcoidosis studies [15, 29-31].

Disease specific sarcoidosis questionnaires seem to be insufficient. The insufficiency first refers to non-English speaking populations; translation and validation studies for their use in other populations are lacking.

The first disease-specific sarcoidosis questionnaire (SHQ) was published in 2003. However, the development and validation study was performed in predominantly (80%) African-American patients [15]. Since that time the first translation-validation study of SHQ in different patients' population was published in 2012 performed in New Zealand [32]. However, the New Zealand patient population is also English-speaking population, although the languages do differ.

There is yet another obstacle in conducting health status studies in non-English speaking populations. The translation-validation process in these countries is one more step to perform, and this is extremely important for all further steps.

In general, health status is a broad concept that is influenced by numerous factors including even extra pulmonary problems. In this study KSQ translated version shows significant correlation with other HS questionnaires already used in sarcoidosis, i.e., SGRQ and 15D (**Table 1**).

In this study we wanted to evaluate the new sarcoidosis specific questionnaire, first validated in UK patients' population in 2012 [7]. After performing the translation process further analyses showed that the translated version of the KSQ showed high internal reliability, similar to the original KSQ version (**Table 2).**

Patients with chronic sarcoidosis (112 pts) had in general lower KSQ scores. Significantly lower scores in chronic sarcoidosis were: lung score, skin and eye score; also, GHS Eye score, GHS Lung, Skin score and GHS Lung Skin Medication (**Table 4).**

For patients with follow up period more than 5 years, clinical outcome status (COS) was analyzed as they were classified into 9 categories (**Table 5**).

In the light of the COS categories, that incorporates many components of sarcoidosis out of which the current or past need for systemic therapy, it was not surprising to realize that

KSQ Medication score correlated with the COS categories; the correlation was negative with p=0.002 (**Table 6**)

In a validation study performed by authors from New Zealand, SHQ scores correlated poorly with lung function parameters [32]. In our study KSQ scores significantly correlated with lung function parameters (considering both spirometry and diffusing capacity). This is in tune with lung involvement of the disease and consecutive airway obstruction findings in sarcoidosis (**Table 6**).

The fact that sarcoidosis involves lung parenchyma, thus affecting blood-gas exchange on the basic levels of lung parenchyma explains good correlation and the advantage of KSQ and its translation version in discovering this fundamental lesion leading to diffusing capacity impairments.

KSQ translated version showed significant correlation with MRC scores, the finding that is even more significant when considering that the MRC addresses the level of activities that leads to dyspnea [34].

The DAL measures the ability to perform various daily activities such as eating, dressing, bathing, using the lavatory, walking, going to the market and climbing the stairs.

The correlation of KSQ translated version with this activity questionnaire is statistically significant in all KSQ domains. Fatigue is a common symptom of sarcoidosis and measuring it is a unique task when speaking about quality of life and /or health status in this patient` population. It is an outstanding advantage of KSQ that can reliably measure this sensation.

However, in our opinion further studies are necessary to distinguish the components of fatigue (mental or physical) in correlation with KSQ.

KSQ has been validated in both German and Dutch languages [35,36]. The authors from the Netherlands have shown good internal consistency for all KSQ, and intraclass correlation coefficients and Bland-Altman plots showed good repeatability of the KSQ, therefore the test was successfully validated for Dutch language [35]. The German version required the reduction of models, from original 29 to 24, in order to fulfil the requirements of the Rasch model; however, the original 29 version can still be used to compare data to international version [36]. Both versions have not been compared to other, previously validated, questionaries, same as the Serbian version.

Conclusion

Translated version of the King’s Sarcoidosis Questionnaire has fulfilled all key messages the original KSQ has. It is a brief tool reliable to assess the health status of patients with sarcoidosis.

The King’s Sarcoidosis Questionnaire is a validated tool suitable for clinical and research use in non-English speaking population. The King’s Sarcoidosis questionnaire can, not only be used as an important measure tool in the evaluation of therapies and clinical outcome in sarcoidosis, but can be used to standardize and compare data internationally, especially when versions in other languages are being developed.

REFERENCES

1. Hunninghake GW, Costabel U, Ando M et al. ATS/ERS/WASOG statement on sarcoidosis. American Thoracic Society/European Respiratory Society/World Association of Sarcoidosis and other Granulomatous Disorders. Sarcoidosis Vasc Diffuse Lung Dis 1999; 16:149–73.
2. Baughman RP, Culver DA, Judson MA. A Concise Review of Pulmonary Sarcoidosis. Am J Respir Crit Care Med 2011; 183:573–581.
3. Cox CE, Donohue JF, Brown CD et al. Health-related quality of life of persons with sarcoidosis. Chest 2004; 125:997–1004.
4. Drent M, Lower E. De Vries J. Sarcoidosis-associated fatigue. Eur Respir J 2012 ; 40:255–263.
5. De Kleijn WPE, Drent M, .Vermunt JK, Shigemitsu H, De Vries J. Types of fatigue in sarcoidosis patients. Journal of Psychosomatic Research 2011; 71:416–422.
6. Cox CE, Donohue JF, Brown CD et al. The Sarcoidosis Health Questionnaire: a new measure of health-related quality of life. Am J Respir Crit Care Med 2003; 168:323–9.
7. Patel AS, Siegert RJ, Creamer D, Larkin G, M Maher TM, Renzoni EA, Wells AU, Higginson IJ, Birring SS. The development and validation of the King's Sarcoidosis Questionnaire for the assessment of health status. Thorax 2013; 68(1):57–65.
8. Judson MA, Baughman RP, Teirstein AS et al. Defining organ involvement in sarcoidosis: the ACCESS proposed instrument. ACCESS Research Group. A Case Control Etiologic Study of Sarcoidosis. Sarcoidosis Vasc Diffuse Lung Dis 1999; 16:75–86.
9. Miller MR, Hankinson J, Brusasco V, et al. Standardisation of spiromerty. ATS/ERS Task Force: Standardization of Lung Function Testing. Eur Respir J 2005; 26:319-338.
10. Sintonen H, Pekurinen M. A fifteen-dimensional measure of health-related quality of life (15D) and its applications. In:Walker SR, Rosser R, editors. Quality of life assessment: key issues in 1990s. Dordrecht: Kluwer Academic Publishers; 1993. p. 185-95.
11. Jones PW, Quirk FH, Baveystock CM, et al. A self-complete measure of health status for chronic airflow limitation. The St George's respiratory questionnaire. Am Rev Respir Dis 1992; 145:1321-7.

12. Sintonen H. The 15D-measure of health-related quality of life: I: reliability, validity and sensitivity of its health state descriptive system: working paper 41. Melbourne, Australia: National Centre for Health Program Evaluation; 1994.
13. Sintonen H. The 15D-measure of health-related quality of life II: feasibility, reliability and validity of its valuation system: working paper 42. Melbourne, Australia: National Centre for Health Program Evaluation; 1994.
14. Gvozdenovic B, Milosevic S, Vukovic M, et al. ‘‘15D’’ and ‘‘MiniAQLQ’’ – validity of the Serbian version of the questionnaires for measurement of quality-of-life e use in bronchial asthma. Opsta medicina 2001; 7:21-36.
15. Chang JA, J. R Curtis, Patrick DL, Ragh G. Assessment of Health-Related Quality of Life in Patients with Interstitial Lung Disease. CHEST 1999; 116:1175–1182.
16. Medical Research Council. Questionnaire on respiratory symptoms: instructions to interviewers. MRC committee on environmental and occupational health. London: MRC; 1986.
17. Stewart AL, Ware JE, Brook RH. Advances in the measurement of functional status: construction of aggregate indexes. Med Care 1981; 19:473-88.
18. Stewart AL, Ware JE, Brook RH. Advances in the measurement of functional status: constructioon of aggregate indexes. Med Care 1981; 19:473-88
19. Gvozdenovic BS, Mihailovic-Vucinic V, Ilic-Dudvarski A, Zugic V, Judson MA. Differences in symptom severity and health status impairment between. patients with pulmonary and pulmonary plus extrapulmonary sarcoidosis. Respir Med 2008; 102:1636–1642.
20. Michielsen HJ, De VJ, Van Heck GL. Psychometric qualities of a brief self-rated fatigue measure: the fatigue assessment scale. J Psychosom Res 2003; 54:345–52.
21. De Vries J,Michielsen H, Van Heck GL, et al.Measuring fatigue in sarcoidosis: the Fatigue Assessment Scale (FAS). Br J Health Psychol 2004; 9:279–91.
22. Baughman RP, Nagai S, Balter M, et al. Defining the clinical outcome status (COS) in sarcoidosis: results of WASOG Task Force. Sarcoidosis Vasc Diffuse Lung Dis 2011; 28:56-64 .
23. Wirnsberger RM, Drent M, Hekelaar N, et al. Relationship between respiratory muscle function and quality of life in sarcoidosis. Eur Respir J 1997; 10:1450–1455.
24. De Vries J, Drent M. Quality of life and health status in sarcoidosis: a review. Semir Respir Crit Care Med 2007; 28(1):121-7.

25. Hoitsma E, Marziniak M, Faber CG, et al. Small fiber neuropathy in sarcoidosis. Lancet 2002; 359:2085–2086.
26. Wirnsberger RM, De Vries J, Wouters EF, Drent M. Clinical presentation of sarcoidosis in the Netherlands: an epidemiological study. Neth J Med 1998; 53:53–60.
27. Drent M, Wirnsberger RM, Breteler MH, Kock LM, De Vries J, Wouters EF. Quality of life and depressive symptoms in patients suffering from sarcoidosis. Sarcoidosis Vasc Diffuse Lung Dis 1998; 15:59–66.
28. Wirnsberger RM, De Vries J, Breteler MH, Van Heck GL, Wouters EF, Drent M. Evaluation of quality of life in sarcoidosis patients. Respir Med 1998; 92:750–756.
29. Guyatt GH, Berman LB, Townsend M, Pugsley SO,Chambers LW. A measure of quality of life for clinical trials in chronic lung disease. Thorax 1987;42:773–778
30. Guyatt GH, Townsend M, Keller J, Singer J, Nogradi S. Measuring functional status in chronic lung disease: conclusions from a randomised control trial. Respir Med 1989; 83:293–297.
31. Wijkstra PJ, Ten Vergert EM, Van Altena R, et al.Reliability and validity of the chronic respiratory questionnaire (CRQ). Thorax 1994; 49:465–467.
32. De Boer S, Wilsher ML. Validation of the Sarcoidosis Health Questionnaire in a non-US population. Respirology 2012; 17: 519–524.
33. Mihailovic-Vucinic V, Zugic V, Videnovic-Ivanov J. New observations on pulmonary function changes in sarcoidosis. Curr Opin Pulm Med 2003; 9: 436-441.
34. Huijnen B, van der Horst F, van Amelsvoort L, et al. Dyspnea in elderly family practice patients. Occurrence, severity, quality of life and mortality over an 8-year period. Fam Pract 2006; 23:34-9.
35. Van Manen MJ, Wapenaar M, Strookappe B, et al. Validation of the King's Sarcoidosis Questionnaire (KSQ) in a Dutch sarcoidosis population. Sarcoidosis Vasc Diffuse Lung Dis. 2016 Mar 29;33(1):75-82. PMID: 27055839.
36. Farin E, Heyduck K, Frye BC et al. Translation and psychometric properties of the King's Sarcoidosis Questionnaire (KSQ) in German language. Health Qual Life Outcomes. 2019 Apr 11;17(1):62. doi: 10.1186/s12955-019-1131-z. PMID: 30975148; PMCID: PMC6460543.

Table 1. Correlation between KSQ scores and other questionnaires

	SGRQ - SYMPTOMS	***SGRQ - ACTIVITY***	***SGRQ - IMPACTS***	***SGRQ - TOTAL SCORE***	***Score DAL***	***Score MRC***	***FAS total***	***Score15D***
GHS score								
r	-0.523	-0.623	-0.588	-0.663	-0.668	-0.567	-0.739	-0.699
p	<0.001	<0.001	<0.001	<0.001	<0.001	<0.001	<0.001	<0.001
Lung score								
r	-0.576	-0.704	-0.677	-0.751	-0.742	-0.623	-0.688	-0.623
p	<0.001	<0.001	<0.001	<0.001	<0.001	<0.001	<0.001	<0.001
Med score					ns	ns		
r	-0.350	-0.175	-0.303	-0.284			-0.292	-0.284
p	<0.001	0.036	<0.001	0.001			<0.001	0.001
Skin score	ns	ns		ns	ns	ns		
r			-0.157				-0.290	-0.172
p			0.051				<0.001	0.032
Eyes score								
r	-0.382	-0.400	-0.405	-0.445	-0.380	-0.410	-0.538	-0.574
p	<0.001	<0.001	<0.001	<0.001	.002	<0.001	<0.001	<0.001
GHS lung score								
r	-0.579	-0.698	-0.664	-0.743	-0.747	-0.621	-0.767	-0.714
p	<0.001	<0.001	<0.001	<0.001	<0.001	<0.001	<0.001	<0.001
GHS skin score								
r	-0.512	-0.594	-0.580	-0.644	-0.646	-0.535	-0.743	-0.690
p	<0.001	<0.001	<0.001	<0.001	<0.001	<0.001	<0.001	<0.001
GHS eyes score								
r	-0.512	-0.595	-0.558	-0.633	-0.561	-0.554	-0.736	-0.740
p	<0.001	<0.001	<0.001	<0.001	<0.001	<0.001	<0.001	<0.001
GHS LS score								
r	-0.560	-0.668	-0.650	-0.721	-0.715	-0.591	-0.773	-0.709
p	<0.001	<0.001	<0.001	<0.001	<0.001	<0.001	<0.001	<0.001
GHS LM score								
r	-0.579	-0.646	-0.656	-0.713	-0.746	-0.576	-0.744	-0.689
p	<0.001	<0.001	<0.001	<0.001	<0.001	<0.001	<0.001	<0.001
GHS SM score								
r	-0.513	-0.526	-0.567	-0.604	-0.597	-0.472	-0.705	-0.651
p	<0.001	<0.001	<0.001	<0.001	<0.001	<0.001	<0.001	<0.001
GHS LMS score								
r	-0.555	-0.611	-0.636	-0.683	-0.710	-0.546	-0.745	-0.686
p	<0.001	<0.001	<0.001	<0.001	<0.001	<0.001	<0.001	<0.001

GHS- General Health Status, LS- Lung Skin, LM- Lung Medication, SM- Skin Medication, LSM- Lung Skin Medication, ns- non significant, r- Spearman correlation coefficient, SGRQ- Saint George Respiratory Questionnaire, DAL- Daily Activity List, MRC- Medical Research Council dyspnea scale, FAS- Fatigue Assessment Scale

Table 2. Internal reliability for KSQ translated version

KSQ modules					
	General health status	***Lung***	***Skin***	***Eye***	***Medication***
Number of items	10	6	3	7	3
Cronbach's α coefficient (Original KSQ)	0.93	0.86	0.84	0.88	0.70
Cronbach's α coefficient (Translated version of KSQ)	0.90	0.91	0.92	0.91	0.67

KSQ- Kings sarcoidosis questionnaire

Table 3. Baseline characteristics of the study participants

Variables	**N (%)**
Age (years), mean±sd (95% Confidence Interval)	49.67±11.12 (47.83-51.56)
Gender n (%)	
Female	107 (67.3)
Male	52 (32.7)
Smoking n (%)	
Non smoker	113 (71.1)
Ex smoker or smoker	46 (28.9)
Stage of the lung disease n (%)	
0	43 (27.2%)
1	69 (43.0%)
2	37 (23.4%)
3	10 (6.3%)
Course of sarcoidosis n (%)	
Acute	47 (26.9%)
Chronic	112 (70.4%)
Extrapulmonary sarcoidosis n (%)	
Yes	45 (28.3%)
No	114 (71.7%)
Different organs involvement n (%)	
Skin	8 (5.0%)
Eyes	3 (1.9%)
Liver	3 (1.9%)
Lymph node	6 (3.8%)
Spleen	4 (2.5%)
Heart	2 (1.2%)
CNS	19 (12%)
Medication n (%)	
No therapy	71 (44.7%)
Prednisone	73 (45.9%)
Methotrexat	5 (3.1%)
Prednisone and Methotrexat	10 (6.3%)
sACE U/L, mean±sd (95% Confidence Interval)	51.32± 27.45 (46.61-56.03)
Lung function, mean±sd (95% Confidence Interval)	
FVC (liter)	3.71±1.08 (3.49-3.84)
FVC (%)	105.50±16.94 (102.89-108.17)
FEV_1 (liter)	2.90±0.90 (2.73-3.02)
FEV_1 (%)	98.44±18.93 (94.87-101.15)
FEV_1/FVC	77.45±10.26 (75.93-79.30)
PEF (liter)	7.41±2.54 (7.04-7.86)
PEF (%)	102.69±21.62 (99.94-106.74)
DCo	8.02±5.88 (6.99-9.10)
DCo (%)	84.36±15.86 (81.51-87.01)

KCo	1.47±0.21 (1.44-1.52)

CNS- central nervous system, ACE- angiotensin converting enzyme, FVC- functional vital capacity, FEV_1- forced expiratory volume in fist second, PEF- peak expiratory flow, DCo- diffusion lung capacity for carbon-monoxide, KCo- Carbon monoxide transfer coefficient

Table 4. KSQ scores in patients with different clinical course of sarcoidosis

	N	***mean±sd***	***p***
GHS score			
Acute	46	66.9±21.3	0.213
Chronic	112	62.1±23.6	
Lung score			
Acute	46	75.8±19.8	0.013
Chronic	112	66.2±25.2	
Medication score			
Acute	46	73.8±17.6	0.124
Chronic	99	67.9±26.7	
Skin score			
Acute	45	91.0±18.4	0.012
Chronic	110	81.3±27.8	
Eyes score			
Acute	45	83.4±18.8	<0.001
Chronic	110	68.4±26.7	
GHS lung score			
Acute	46	70.5±18.8	0.058
Chronic	112	63.7±23.0	
GHS skin score			
Acute	46	70.5±18.9	0.045
Chronic	112	63.7±23.0	
GHS Eyes score			
Acute	45	73.9±15.7	0.003
Chronic	110	64.4±22.1	
GHS Lung Skin score			
Acute	45	74.1±16.2	0.015
Chronic	110	66.4±21.4	
GHS Lung Medication score			
Acute	44	70.5±16.7	0.047
Chronic	99	63.8±21.7	
GHS Skin Medication score			
Acute	43	72.8±15.3	0.049
Chronic	98	66.6±20.0	
GSH Lung skin medication score			
Acute	43	73.7±14.8	0.016
Chronic	98	62.2±20.5	

GHS- General health status

Table 5. Clinical outcome status and KSQ

<table>
<tr><td colspan="6">Sarcoidosis patients observed during five years (74 patients)</td></tr>
<tr><td>Resolved</td><td>Minimal Disease</td><td colspan="4">Persistent sarcoidosis</td></tr>
<tr><td>COS 1 Never treated</td><td>COS 3 Never treated</td><td colspan="2">No current therapy</td><td colspan="2">Current therapy</td></tr>
<tr><td>COS 2
No therapy > One year
2pts</td><td>COS 4
No therapy > One year
9pts</td><td>COS 5
Never treated</td><td>COS 6
No therapy > One year
21pts</td><td>No worsening prior year</td><td rowspan="3">COS 9
Worsening in prior year
14pts</td></tr>
<tr><td colspan="4" rowspan="2"></td><td>COS 7
Asymptomatic
5pts</td></tr>
<tr><td>COS 8
Symptomatic
23pts</td></tr>
</table>

COS- clinical outcome status

Table 6. Correlation between lung function parameters, clinical outcome of sarcoidosis and KSQ

KSQ	***Lung function parameters***				
	FVC (%)	***FEV$_1$ (%)***	***PEF (%)***	***DLco (%)***	***Clinical outcome of sarcoidosis***
GHS score	ns	ns			ns
r			0.252	0.289	
p			0.001	<0.001	
Lung score	ns				
r		0.168	0.290	0.343	-0.236
p		0.034	<0.001	<0.001	0.042
Medication score	ns	ns	ns	ns	
r					-0.369
p					0.002
Skin score	ns	ns	ns	ns	ns
r					
p					
Eye score	ns	ns			ns
r			0.177	0.230	
p			0.027	0.004	
GHS Lung score	ns	ns			
r			0.285	0.332	-0.256
p			<0.001	<0.001	0.027
GHS Skin score	ns	ns			
r			0.223	0.305	-0.235
p			0.005	<0.001	0.037
GHS Eye score	ns	ns			ns
r			0.259	0.315	
p			0.001	<0.001	
GHS Lung Skin score	ns	ns			
r			0.259	0.345	-0.245
p			0.001	<0.001	0.037
GHS Lung Medication score	ns	ns			
r			0.251	0.380	-0.286
p			0.002	<0.001	0.020
GHS Skin Medication score	ns	ns			
r			0.185	0.343	-0.289
p			0.027	<0.001	0.020
GHS Lung Skin Medication score	ns	ns			
r			0.227	0.387	-0.283
p			0.007	<0.001	0.002

KSQ- Kings sarcoidosis questionnaire, GHS- General health status, ns- non significant, r- Pearson correlation coefficient, FVC- functional vital capacity, FEV_1- forced expiratory volume in fist second, PEF- peak expiratory flow, DL_{CO}- diffusion lung capacity for carbon-monoxide

Questionnaire

Click here to access/download
Supporting Information
KSQ- serbian.docx
