# Supplementary material for: King’s Sarcoidosis Questionnaire (KSQ) – Validation study in Serbian speaking population of sarcoidosis patients
Source: PLoS One. 2023 Sep 5;18(9):e0273126. doi: 10.1371/journal.pone.0273126 (PMC10479938; doi:10.1371/journal.pone.0273126)
Supplement: Attachment — Submitted filename: 12-11-2022 Reviewer-Anonymous (KSQ Serbian version).pdf [file pone.0273126.s007.pdf]

Dear Authors,

This paper investigated the PLOS ONE manuscript "King's Sarcoidosis Questionnaire (KSQ) – Validation study in Serbian speaking population of sarcoidosis patients."

The manuscript revision has been improved; all the queries have been answered appropriately. The authors had already responded to the main concerns and relevant issues. I found this work to be persuasive and consistent with scholarly intent and intellectual passion to contribute to supporting sarcoidosis patients in Serbia.

One recommendation for further study would be to utilize the treatment (before-after), which can strengthen your work and benefit sarcoidosis patients in Serbia.

I recommended this work for publication or no revision.

Thank you very much

Reviewer
