# Supplementary material for: King’s Sarcoidosis Questionnaire (KSQ) – Validation study in Serbian speaking population of sarcoidosis patients
Source: PLoS One. 2023 Sep 5;18(9):e0273126. doi: 10.1371/journal.pone.0273126 (PMC10479938; doi:10.1371/journal.pone.0273126)
Supplement: Attachment — Submitted filename: Review King (KSQ Serbian).docx [file pone.0273126.s005.docx]

**Review King’s Sarcoidosis Questionnaire (KSQ) -Validation study in non-English speaking population of sarcoidosis patients**

**Comment to authors**

This article provides the validation results of the King’s Sarcoidosis Questionnaire (KSQ) in the non-English speaking population of sarcoidosis patients. The manuscript has limitations on language usage as there were a lot of non-academic and awkward sentences. It would be good if the authors could revise the manuscript to make it more precise and constructive. In my opinion, this paper requires significant revision. I hope my comments and suggestions below are helpful.

- The authors should reconsider the title of this research to be specific to the sample of the current study (Serbian) instead of using non-English speaking.
- Abstract; no need to mention another questionnaire that was not used in the current study.

**Introduction**

- The Introduction is too short. The authors should provide more rationale for research gap.
- A comprehensive literature review should be provided to reveal the correlation between the quality of life and sarcoidosis patients’ life
- Please provide the rationale for choosing the King’s Sarcoidosis Questionnaire (KSQ) over the Sarcoidosis Health Questionnaire.
- Please add more information on previous cross-cultural studies that examined the psychometric properties of the KSQ.

**Methods**

- Please give detail on the inclusion and exclusion criteria used. It’s not clear in the methods.
- Please provide more information on sample recruitment and the rational of enrolling samples 18 years and older.
- Please revise the Methods section to be more structured and clearer, particularly on Questionnaire
- ‘Questionnaire’ should be on the sub-heading not ‘ questionnaire used in this study’
- Please provide psychometric properties, i.e. internal consistency of all questionnaires used in the current study
- In the back translation process, two bilinguals who expertise both in English and Serbian are needed, not only two mother tongue experts
- No need to mention the full name of the person who scored the KSQ on the manuscript
- In my opinion, the authors should provide only the Item-Objective Congruence (IOC) results instead of giving all doctors’ comments on the manuscript

**Statistical analysis**

- Please elaborate statistical analysis more precise and clearly
- Please provide how missing data was handled
- Description of results of normality of distribution should be evident in the section

**Results**

- There are many questionnaires used in the current study and also an abbreviation, therefore the authors should consider reporting results more precise and clearly by using table

**Discussion**

- The first paragraph of the Discussion should be moved to Introduction
- No need to repeat all the results of the discussion.
- The discussion needs to improve and add information on the strengths and limitations of the study.
- Please add the information about the clinical application of this study.
- As this current study examines convergent validity and internal consistency, it could not be concluded that the KSQ is a reliable and valid tool. The findings should be interpreted with caution regarding the limitation. Other psychometric properties, e.g. test-retest reliability, criterion validity, and construct validity of the KSQ should also be examined.
