# Supplementary material for: King’s Sarcoidosis Questionnaire (KSQ) – Validation study in Serbian speaking population of sarcoidosis patients
Source: PLoS One. 2023 Sep 5;18(9):e0273126. doi: 10.1371/journal.pone.0273126 (PMC10479938; doi:10.1371/journal.pone.0273126)
Supplement: Attachment — Submitted filename: Response to Reviewers.docx [file pone.0273126.s006.docx]

To whom it may concern,

We have taken into consideration the points raised by the academic editor and reviewers, and have corrected accordingly.

- The majority of the article has been rewritten, as to improve the quality of language.
- The title of the article has been changed, as to be more specific (from “non-English” to “Serbian”).
- We have removed the other questionnaire that was not used in the study from the Abstract.
- We have expanded the introduction as to emphasize the need for a quality-of-life questionnaire.
- The ethical aspect, as well as the inclusion and exclusion criteria have been expanded upon in material and methods section.
- Other questionnaires that have been used for comparison have been explained as well as rationale behind using them in the material and methods section.
- We have removed the results from the discussion section.
- The limitations of the study have been noted in the discussion section.
- Clinical application of the questionnaire has been explained in the conclusion.
- The versions of KSQ in other languages were discussed, and the rationale for using the KSQ over Sarcoidosis Health Questionnaire was explained.

We hope that our changes to the article will suffice, and that our altered article will meet the criteria for publication.

The autors
