# Supplementary material for: King’s Sarcoidosis Questionnaire (KSQ) – Validation study in Serbian speaking population of sarcoidosis patients
Source: PLoS One. 2023 Sep 5;18(9):e0273126. doi: 10.1371/journal.pone.0273126 (PMC10479938; doi:10.1371/journal.pone.0273126)
Supplement: Attachment — Submitted filename: Response to Reviewers.docx [file pone.0273126.s008.docx]

To whom it may concern,

We are grateful for positive responses on our previously implemented corrections. Having taken into consideration the points raised by the reviewers, we have corrected the paper accordingly.

- The introduction section of the abstract is shortened, as suggested.
- We have removed the mention of the other questionnaire (Sarcoidosis health questionnaire) in the previous version of the paper
- The Ethic board of Clinic of pulmonology, University Clinical Center of Serbia, Belgrade, Serbia had given the permission to conduct this study
- The rationale behind other questionnaires was explained
- The results generated by the study were clarified, and another table was provided in order to explain further

We hope that our changes to the article will suffice, and that our altered article will meet the criteria for publication.

The autors
