# Supplementary material for: King’s Sarcoidosis Questionnaire (KSQ) – Validation study in Serbian speaking population of sarcoidosis patients
Source: PLoS One. 2023 Sep 5;18(9):e0273126. doi: 10.1371/journal.pone.0273126 (PMC10479938; doi:10.1371/journal.pone.0273126)
Supplement: Attachment — Submitted filename: Response to Reviewers.docx [file pone.0273126.s009.docx]

To whom it may concern,

We are grateful for your suggestions regarding our paper titled “King’s Sarcoidosis Questionnaire (KSQ) – Validation study in Serbian speaking population of sarcoidosis patients”. We have tried to respond to all of the suggestions, and have noted the changes in the manuscript, as required:

1. The rationale behind the need is now in Introduction section, as suggested.
2. COS groups are now presented in a table
3. As both of the original authors have read and approved the Serbian version, back translation was not performed prior to cognitive interviewing
4. The rating of 3 by the last participant was acknowledged and explained upon in the text
5. Sample size was elaborated on in the results
6. Our five components correspond to the original five modules of the KSQ, which is now noted in the paper
7. Internal consistency cronbach’s alpha is now noted in the paper.

We hope that our changes are satisfactory, and are grateful that you were able to provide a new Reviewer, after the previous one became unavailable. Looking forward for a positive response.

Sincerely,

Authors
